# Reducing Lead on the Landscape: Anticipating Hunter Behavior in Absence of a Free Nonlead Ammunition Program

**DOI:** 10.1371/journal.pone.0128355

**Published:** 2015-06-26

**Authors:** Loren Chase, Michael J. Rabe

**Affiliations:** 1 Social Science Research Program, Arizona Game and Fish Department, Phoenix, Arizona, United States of America; 2 Nongame Branch-Wildlife Management Division, Arizona Game and Fish Department, Phoenix, Arizona, United States of America; INIBIOMA (Universidad Nacional del Comahue-CONICET), ARGENTINA

## Abstract

Lead is a neurotoxin that has been documented to affect many forms of wildlife, and has been identified as a limiting factor in a population of California Condors in Northern Arizona. The Arizona Game and Fish Department provides vouchers for free nonlead ammunition to hunters selected to hunt within the distribution of California Condors, with the intention of having fewer lead-laden offal piles available to California Condors. Although wildlife agencies may reasonably assume voucher programs motivate hunters into choosing nonlead ammunition, the lead reduction efforts attributable to the voucher program has not been empirically quantified. Our intention was to compare a control group of hunters to a treatment group of hunters within California Condor occupied areas. Both groups received educational materials regarding the deleterious effects of lead, but the treatment group also received a voucher for a free initial box of ammunition. About half of the control group used nonlead ammunition, compared to about three-fourths of the treatment group. Prominent barriers to adoption of nonlead ammunition included a general difficulty of obtaining it, obtaining it in the desired caliber, and its costliness. Frequently mentioned motivations for using nonlead was the exhortation to use it by the Department, and the desire to aid California Condor recovery by hunters. The disparate compliance rates found herein confirm and quantify the success of nonlead ammunition voucher programs, but underscore the importance of working to increase the supply of nonlead ammunition with the end of facilitating its procurement and reducing its cost.

## Introduction

Lead is a naturally occurring element that is a neurotoxin to animals. Although lead poisoning has been documented in mammals [1], much of the literature regarding ingested lead and wildlife focuses on avian species [2]. Waterfowl are particularly susceptible to lead poisoning [3, 4], although passerines [5] and game birds [6, 7, 8] have also been documented to have lead toxicosis in individual animals. To a greater extent, scavenger birds such as Ravens [9] and Turkey Vultures [10] are susceptible to lead poisoning. Further, raptors have also been documented to have ingested lead and subsequently developed lead toxicosis [11, 12]. Although there is ample literature documenting lead poisoning in individual animals, or even in subpopulations, there is debate as to whether these incidences affect these wildlife species at the population level. One species wherein lead toxicosis has been documented to have deleterious effects at the population levels is the California Condor [13, 14].

For myriad reasons, the California Condor (*Gymnogyps californianus*) population significantly declined to a point where in 1982 only 23 individuals remained in the wild [15,16]. Because of the eminent threat to the species, the entire wild population was captured in 1987 and a captive breeding program was initiated to recover the birds [15]. In the ensuing decade, the captive breeding program produced sufficient numbers of birds to reintroduce populations in several areas of California, Arizona, and Mexico.

In spite of the success of the captive breeding program, some California Condors released into the wild have died due to lead toxicosis primarily derived from spent ammunition. This mortality due to lead ingestion is a major impediment to California Condor recovery in the West [17,18]. Since the initiation of the captive breeding program, 135 California Condors have died. Lead toxicosis is the leading cause of diagnosed mortality among juveniles and adults released into the wild (proportional mortality rate at 26% and 67% respectively; [16]). Some dispute the lead responsible for the deaths of California Condors originates from spent ammunition [19]; however, lead was present in 90% of offal piles in rifle-killed deer [20]. Observations of California Condors in proximity to deer remains [21] and spikes in lead levels in California Condor blood serum during and immediately following deer hunting seasons [16] support the conclusion that lead ammunition is a major source of lead ingestion by California Condors [22]. Although governments from 29 countries have placed regulations on lead ammunition [23], many state wildlife agencies (agencies) and hunter groups are advocating for voluntary measures to reduce lead available in California Condors.

Notwithstanding the deleterious effects of lead toxicosis, the California Condor program continues to reintroduce populations to portions of former home ranges in Arizona under section 10(j) of the Endangered Species Act. The 10(j) area encompasses portions of northern Arizona, southern Utah, and extreme southwest Nevada. In Arizona, California Condors were reintroduced near Vermilion Cliffs National Monument beginning in 1996. By July 2013, 139 California Condors had been reintroduced to Arizona, and reproduction from those birds resulted in 22 wild-hatched chicks [24](USFWS, 2013). Since the 1996 reintroduction, 84 California Condors have died, approximately 54% of which are attributable to lead (A. Zufelt, personal communication, December 3, 2014). Since 2005, nearly half of the California Condors sampled in Arizona are annually treated for lead through chelation (C. Parish, personal communication, January 23, 2014). Lead in the form of fragments, shot, or whole bullets have been identified in the digestive tract of 19 dead California Condors, out of the 28 necropsied on suspicion of lead toxicosis (C. Parish, personal communication, January 23, 2014; [25]). Given the cyclical peak in lead toxicosis during and immediately following hunting seasons [21], along with the finding that 50% of the known causes of death in the 10(j) area is attributed to lead toxicosis, lead ammunition from hunter-killed game has been identified as a major source of mortality in the Arizona/ Southern Utah California Condor population.

Arizona Game and Fish Department (AGFD) implemented a multifaceted lead-reduction program in an effort to reduce further deaths of California Condors. The program includes annual outreach to big game hunters selected to hunt within the Arizonan portion of the California Condor range using brochures, mailings, coordination with hunter constituency groups, and personal contact with hunters in the field. Messages from the AGFD center upon changes hunters can make to reduce lead available to California Condors, including using non-lead alternatives.

There has been a general reluctance of some hunters to adopt new ammunition, citing barriers such as cost, performance, and intermittent availability [26–28]. In 2005, to assist hunters in overcoming these obstacles, AGFD began providing free nonlead ammunition to hunters of some areas (units 12 and 13) within California Condor habitat. This program has resulted in as much as 77% (2011) of hunters using nonlead ammunition. In the same year, other hunters took alternative steps to reduce lead available to California Condors (i.e., removing offal piles from the field, burying offal piles), for a total of 90% of hunters taking some measure to reduce lead available to California Condors. These voluntary lead reduction programs have been effective at reducing lead within the foraging range of California Condors [29]. Additional simulation models indicate voluntary lead reduction programs would be effective at reducing California Condor exposure to lead in other areas as well [30, 31]. However, uncertain funding sources, increased ammunition costs, and the expanding distribution of California Condors may all be factors that affect the long-term sustainability of free ammunition programs, despite the success of the voluntary program in Arizona.

Voluntary participation is thought to be a preferred method to reduce the lead available to California Condors for several reasons. Primarily, volunteer programs require multiple groups to cooperate, producing social capital, which yields greater civic engagement [32]. Civic engagement is the keystone to creating hunting and angling laws through transparent public processes, a central tenet in the North American Model of Wildlife Conservation [33]. Additionally, hunters, who frequently self-identify as the original conservationists, may be alienated if mandatory bans were instituted, similar to the estrangement that resulted from the ban on lead-based ammunition for waterfowl in the early 1990s. Declines in hunting participation similar to those seen in the 1990s would diminish Federal Aid in Wildlife Restoration Funds (a federal excise tax on hunting and shooting equipment, commonly known as the Pittman-Robertson Act of 1937) resulting in reduced conservation funding for all species of wildlife. Further, enforcement of a regulatory ban on lead ammunition would be problematic since there is currently no established method to field test bullet composition.

### Study purpose

This research aims to understand and anticipate hunter behavior associated with reducing spent lead ammunition on the landscape. Specific objectives were to (1) compare usage rates between hunters who received free nonlead ammunition and hunters who did not, (2) identify barriers to using nonlead ammunition, and (3) identify important factors influencing the decision to use nonlead ammunition for hunters who switched in the absence of an incentive. We address these research questions in the form of a quantitative survey of big game hunters in California Condor distributions within Arizona.

## Study Area

Wildlife management units (units) 9 and 10 are located north and west of Flagstaff, Arizona, and south of the Hualapai Reservation and the Grand Canyon National Park. Units 12 and 13 are on the north side of the Hualapai Reservation and the Grand Canyon National Park. Units 9 and 10 make excellent analogs for units 12 and 13 due to their proximity, matched biological conditions, and similar hunting regulations. Hunting opportunities in these areas are allocated through the AGFD draw process (lottery by which hunters are selected to certain hunts), so the names and addresses of the entire sample universe are known. AGFD mailed all individuals drawn for hunting deer and elk in 9, 10, 12, and 13 a physical permit tag accompanied by educational materials discussing the effect of lead on California Condors, as well as an exhortation to use nonlead ammunition. Individuals drawn for hunting in units 12 and 13 also received a voucher for nonlead ammunition, while hunters in 9 and 10 received no vouchers. These conditions were ideal to investigate potential hunter behavior in regards to ammunition choice and lead-reducing activities.

## Methods

### Ethics statement

All data collection associated with this research was conducted with the utmost care for respondents. With each version of the survey sent to participants, a cover letter briefly explained the purpose of the study and served as informed consent. Participants were informed that the study was low risk, participation was voluntary, and they could withdraw at any time. Participants were also guaranteed confidentiality, their responses would never be associated with any identifying information, and that all results would be reported in aggregate. All identifying information was removed prior to data analysis.

### Survey instrument

AGFD developed and pre-tested the survey instrument for question clarity and flow to reduce respondent burden. The survey instrument included three sections (1) behavioral information regarding days afield, hunt success, hunting in the area prior to the 2011 season and use of nonlead ammunition, (2) demographics, and (3) factors that affected their decision to use nonlead ammunition (for hunters who chose to do so) or barriers to using nonlead ammunition (for hunters who did not use nonlead ammunition). Factors influencing the decision to use nonlead ammunition were collected on a 5-point scale of importance, anchored by 1-Not Important at All and 5-Extremely Important. Barriers to using nonlead ammunition were collected on a 5-point agree/disagree scale, ranging from 1-Strongly Disagree to 5-Strongly Agree. AGFD mailed surveys to 1,500 hunters, randomly selected from all hunters chosen to hunt in the fall deer and elk general season in units 9 and 10. Survey data were not collected on hunters in units 12 and 13 as they are extensively monitored via a mandatory check and comprehensive field contact, so their compliance with lead reduction efforts is known.

To minimize nonresponse bias, a Modified Dillman Method [34] was used, consisting of an initial postcard, an initial survey, a reminder postcard for hunters who had not yet returned the surveys, and a second survey sent to those who still failed to respond. Data were collected February through March of 2012 to accommodate all deer and elk hunts occurring during the season, ranging from early September through December. The Statistical Package for the Social Sciences, SPSS/PASW 18.0 was used for all statistical analyses, and statistical significance was designated at a level of *p*<0.05.

## Results

AGFD selected 4,350 deer and elk hunters to hunt in units 9 and 10. From these, a sample of 1,500 hunters were selected to participate in this study, and 980 returned surveys (65% response rate), yielding a confidence level of 95% at a margin of error of 2.76%. Ninety-four percent (*n* = 920) of respondents indicated they hunted in units 9 or 10 and 34% (*n* = 306) successfully harvested game. Many (61%) of the hunters from the 2011 season had hunted in the same area prior to the current year, most of those (76%) within the past two years. There was no difference in harvest success (Χ^2^ = 0.37, *p* = .56, *φ* = .02), age (F = 2.40, *p* = .12, *η* = .05), days afield (F = 0.03, *p* = .87, *η* < .001), days scouting (F = 0.65, *p* = .42, *η* = .001), or prior hunting experience in the area (Χ^2^ = 0.13, *p* = .72, *φ* = .01) between hunters who used or did not use nonlead ammunition.

All hunters in units 9 and 10 received educational materials and a letter from AGFD encouraging the use of nonlead ammunition, yet only 88% (*n* = 788) recalled receiving the information before their hunt. Importantly, of hunters who used nonlead, 94% recalled receiving the materials compared to 82% of hunters who chose not to use nonlead (Χ^2^ = 28.35, *p* < .001, *φ* = .18). Despite receiving the educational materials and a request from AGFD to consider using nonlead ammunition, only half (49%, *n* = 452) of hunters in units 9 and 10 reported using nonlead ammunition for their 2011 hunt, in the absence of nonlead ammunition vouchers. During the same timeframe, 77% of hunters in units 12 and 13 (those that received nonlead ammunition vouchers) reported using nonlead ammunition. The voluntary compliance rate of hunters who received an ammunition voucher was 27.7% higher than hunters who did not receive a voucher; a difference that is strongly statistically and practically significant (*t*_915_ = 16.73, *p* < .001; Cohen’s *d* = 1.106).

The 49% of hunters in units 9 and 10 who used nonlead ammunition were generally satisfied with it, as indicated by the 79% intending to use nonlead ammunition in the future and the 75% who were willing to recommend it to fellow hunters. When asked which factors were important in choosing to use nonlead ammunition on their hunt, the most important factors were the request to use it by AGFD (73% indicating important or extremely important) and a desire to help California Condor recovery (68%; Table 1). Less important factors included using nonlead for its reputation (54%) and ballistic performance (54%). Respondents were also able to supply additional reasons for using nonlead ammunition not captured on the survey. The foremost respondent-volunteered reasons for using nonlead ammunition included the desire to protect animals other than California Condors (e.g.: “less toxic for all scavengers,” “protect other wildlife that feed on carrion”) and concerns regarding human health (e.g.: “safer for consumption,” “toxicity concerns,” and “[I] don’t want to eat lead”). Of all the actions hunters took to reduce lead on the landscape, using nonlead ammunition was the primary action (49%), complemented with removing offal piles or burying offal piles to make them unavailable to California Condors (4.1%).

**Table 1:** Hunter responses to two Factors influencing the decision to use nonlead ammunition and barriers to nonlead ammunition use in Northern Arizonan hunters.

***“What factors were important to you while making your decision to use nonlead*?*”*** (asked only to hunters who used nonlead ammunition (*n* = 452; 49%)							
	**Response Items**					**Scale**	
	**Not important at all**			**Extremely Important**			
	**1** ^**a**^	**2**	**3**	**4**	**5**	**Mean**	**SE**
**Game & Fish asked me to**	6	3	19	34	39	3.98	1.102
**To help California Condor recovery efforts**	6	5	21	28	40	3.90	1.170
**I like the performance of nonlead**	6	6	34	23	31	3.67	1.159
**I read/heard about its performance**	8	7	32	27	27	3.58	1.174
**I already used it**	16	6	24	25	29	3.46	1.381
***What prevented you from using nonlead ammunition in your hunts this year*?** (asked only to hunters who used lead ammunition (*n* = 465; 51%)							
	**Strongly Disagree** ^**b**^	**Disagree**	**Neither**	**Agree**	**Strongly Agree**	**Mean**	**SE**
**It is too difficult to find in stores**	9	10	39	28	14	3.29	1.095
**It costs too much for me to use**	9	14	47	20	10	3.07	1.046
**I’m not convinced lead is an issue**	19	19	35	14	13	2.83	1.254
**It doesn’t perform as well as lead**	7	12	57	13	11	3.09	0.989
**It wasn’t available in my caliber**	16	17	48	11	7	2.75	1.082
**I forgot to use it**	18	12	54	10	5	2.72	1.030

^a^ Five-point importance scale anchored by 1-Not important at all and 5-Extremely important

^b^ Five-point agreement scale of 1-Strongly Disagree, 2-Disagree, 3-Neither, 4-Agree, 5-Strongly Agree

The other 51% of hunters in units 9 and 10 did not use nonlead ammunition for a variety of reasons. Chief among these reasons included the difficulty of finding nonlead ammunition in stores (42%), and the higher expense as compared to lead ammunition (30%). Less important factors included concerns regarding its performance (24%), unavailability in desired calibers (18%), and not remembering to use it (15%; Table 1). These hunters were also able to document self-generated reasons for not using nonlead ammunition. These reasons included not having enough time (e.g.: “[educational materials] sent too late,” “Didn’t have time to resight rifle [to account for the different point of impact of nonlead]”), issues with reloading (e.g.: “Didn’t have time to work up loads,” “[I] reload, it’s hard to find”), unfamiliarity with the issue (e.g.: “did not know,” “never heard of issue relating to condors & lead poisoning”), and not attributing their actions as contributing to the issue (e.g.: “don’t hunt where the problem is,” “hunted in Dec & no condors in our area”).

Hunters that chose not to use nonlead ammunition were asked what would convince them to use nonlead ammunition on their next hunt. A third (33%) indicated if the costs were comparable to traditional ammunition they would use nonlead, 26% would switch to nonlead if their concerns with its ballistic performance were resolved, 25% wanted more information regarding the effect of lead on wildlife before they would switch, 19% said they would use nonlead ammunition if it were provided free of cost, and another 19% indicated they would switch if nonlead ammunition were available in their preferred caliber. Nine percent of hunters that did not use nonlead ammunition, or 4.3% of all hunters, stated that nothing would convince them to use nonlead ammunition. Hunters that did not use nonlead ammunition were also able to state self-generated factors that would motivate them to switch, which included availability (e.g.; “not available in local stores,” “not available at the time,” and “not in stock on what I wanted to shoot”) and a current stockpile of lead ammunition that hunters prefer using before buying new ammunition (e.g.; “have to use supply of lead ammo,” and “After lead supply is gone”). Although all hunters drawn for big game in units 9 and 10 were hunting in areas of California Condor distribution, several hunters did not attribute their actions as contributing to the California Condor issue (e.g.: “Never seen a condor in unit 9 except on Colorado River eating from a garbage can,” “don’t hunt near [the Grand] Canyon,” and “Don’t believe condors get as far south as immediate area of Williams” (a town within, albeit on the periphery, of California Condor range). Hunters that did not switch to nonlead ammunition still showed interest in California Condor conservation, as 21% of successful hunters using lead ammunition removed the offal pile from the field.

## Discussion

Foremost, these research findings attest to the success of nonlead voucher programs in reducing lead from spent ammunition available to California Condors. Hunters who received free nonlead ammunition were much more likely to use nonlead than those hunters who did not receive their first box of ammunition for free (77% and 49% respectively). Barriers to switching to nonlead ammunition continue to be the general difficulty of obtaining it, obtaining it in the desired caliber, and concerns with its ballistic performance. To a lesser degree, hunters’ unawareness of the consequences of their decision to use lead ammunition is also a barrier to California Condor recovery. For hunters who switched to using nonlead ammunition in the absence of an initial free box of ammunition, two prominent motivations for switching included the exhortation to use it by the Department, and the moral obligation to conserve wildlife.

Our research documents the success of nonlead voucher programs as state agencies could expect a marked reduction in compliance rates in the absence of a free nonlead ammunition voucher program, given that 77% of hunters use nonlead ammunition when provided a voucher, versus 49% who use nonlead in areas without such a program. Given there are approximately 1,400 hunters who currently receive the vouchers annually, and the average hunter harvest rates of units 12 and 13, the absence of this program would likely result in an estimated 376 additional lead-laden offal piles. Further, if the voucher program were extended to units 9 and 10 and had successes similar to those already seen in units 12 and 13, the result would be an estimated 1,218 fewer lead-laden offal piles available to California Condors, within their known distribution.

Although these estimates of fewer lead-laden offal piles are encouraging, the estimate of 49% of hunters in units 9 and 10 complying with the request of AGFD to use nonlead ammunition should be considered a ceiling statistic for several reasons. Because the respondents knew AGFD was conducting the survey, some may have been influenced by interviewer bias (altering responses on account of the characteristics of the interviewer) and social-desirability (strategically altering responses to be more congruent with social norms) associated with using nonlead ammunition, though these effects are likely low when using the mail mode of data collection [35]. The 49% compliance estimate may also be high as the response rate for this study was 65%, and, although high for a mail mode of data collection, it may be a reasonable assumption that those who did not heed the exhortation by AGFD to use nonlead ammunition may also have spurned the invitation to participate in a survey on the same topic. However, respondents to earlier waves of the survey did not differ from respondents of later waves (which often are more comparable to survey non-respondents); suggesting this bias may be minimal, though unquantified. Further, throughout the data collection process and in anecdotal conversations with hunters afield, it is apparent that many are unaware which ammunition is actually nonlead. For example, 30% of hunters who claimed to be using nonlead ammunition identified solid copper ammunition as their nonlead ammunition of choice. Yet a portion of hunters who claimed to have used solid copper ammunition actually listed copper-jacketed, lead-core ammunition or reload bullets. Therefore, a portion of hunters who believed they had made the choice to use nonlead ammunition were inadvertently using copper-jacketed ammunition, unaware of the lead core. To remedy the issue of hunter unawareness of lead in their ammunition, instituting universal labeling or added logos to packaging (subject branding) to distinguish nonlead ammunition from lead-based ammunition may facilitate the choice of hunters who voluntarily use nonlead ammunition when in California Condor range.

While there were many impediments that prevented hunters from using nonlead ammunition, its availability, both in sporting goods retail shops and in specific calibers, continues to be a significant hindrance to its use. Agencies can encourage the use of nonlead ammunition by working with retailers to increase the availability of nonlead ammunition, especially in California Condor ranges. Online purchasing options directly from the manufacturer or wholesaler will further allow individuals to obtain their preferred weight and caliber, reducing the burden on retailers to stock nonlead ammunition in multiple weights and calibers. Additionally, if online vendors were eligible to redeem the free ammunition vouchers, hunter compliance would likely increase, as ease of redemption was identified as a compliance barrier by respondent comments to this research effort, and in prior research in the same study area [27].

A portion of hunters remain concerned with the performance of nonlead ammunition, despite growing evidence that lead and nonlead ammunition has increasingly similar ballistic standards [36, 27], have the same effectiveness [37, 38], and the disparity in cost is diminishing when ammunition of similar quality is compared [38]. Agencies may address this issue by emphasizing evidence that supports the fact that nonlead and lead ammunition perform comparably in the field. For instance, there was no statistical or practical difference in harvest success between hunters who did or did not use nonlead ammunition in this study. Further, agencies can improve their efforts by sharing success stories and anecdotal endorsements of hunters who have used nonlead ammunition to harvest game successfully. An additional, yet related, concern of hunters was that nonlead ammunition does not necessarily perform inferiorly to lead ammunition, but performs differently. Several hunters mentioned they would have been willing to use nonlead ammunition had they been asked earlier (to resight rifle scopes to account for the different impact point of nonlead ammunition). Agencies may overcome this obstacle by sending educational materials to hunters further in advance of the hunting season to allow hunters the time to purchase or reload nonlead ammunition, evaluate it, and re-sight their rifle to account for the altered ballistics. Nevertheless, of the hunters who did not use nonlead ammunition, 57% indicated they neither agreed nor disagreed that nonlead performed inferiorly. A portion of these undecided hunters may have fully considered all aspects of the ballistic performance on nonlead ammunition and have decided to they are truly neutral to the topic. However, it is more likely a large majority of these undecided hunters have not considered the topic in sufficient depth to fully form an attitude. Agencies may promote California Condor conservation by endorsing nonlead to hunters who superficially (peripheral cognitive processing) consider this information, in addition to producing authoritative outreach materials and scientifically credible research for those hunters who would like to fully consider issues (central cognitive processing) related to California Condor-ingested lead [39]. Because this is a multifaceted, sociobiological issue with biological, political, social, and economic ramifications, wildlife-associated recreationists ought to recognize their role in the recovery of California Condors [40, 14] and agencies should be using best practices in persuading hunters and anglers to make appropriate conservation choices [41].

Beyond misconceptions of performance, hunters’ unawareness of the consequences of their actions is also a barrier to California Condor recovery. Although all respondents in this study were hunting within California Condor distributions, several hunters expressed the belief that California Condors did not inhabit the area they were hunting. Prior research has suggested if a person believes their action would not result in meaningful change [42,43], are unaware of the consequences of their behaviors, or do not ascribe responsibility of their actions to themselves [44], a person’s behavior may not reflect the attitude they hold regarding the topic. Specifically in this research, hunters chose not switch to nonlead ammunition because they believed they were not contributing to the problem, as they had not personally seen a California Condor or they believed California Condors were not in the area. Because California Condors use units 9 and 10 less frequently than units 12 and 13, this may have also influenced hunter compliance with the voluntary lead reduction measures in these areas. Agencies may reduce lead available to California Condors by educating hunters about the true extent of the distributions of California Condors, the extent of their daily travel, and the ability of California Condors to detect carrion from long distances [45]. Because many hunters may doubt the existence of California Condors in their hunting areas without personal experience, outreach materials should include distribution maps of known California Condor locations to maximize the ascription of personal responsibility.

Finally, a small, but not insignificant, portion of all hunters (4.3%, or 8.6% of hunters who did not use nonlead ammunition) indicated they did not use nonlead ammunition and AGFD could not do anything to convince them otherwise. The content of many of the comments from these hunters suggests they may be fearful that initiatives designed to conserve California Condors are an attempt to incrementally restrict gun use. In this way, support for California Condor recovery originating from organizations known for anti-hunting or anti-gun advocacy may be counterproductive because they exacerbate fears of future firearm regulation. Furthermore, for agencies to maintain credibility, it is essential that they disassociate California Condor conservation via reduction of available lead from social issues associated with rights of gun ownership. To allay some of these fears, agencies may use the verbiage in the Federal 10(j) final rule to assist concerned hunters in cognitively disconnecting these two issues as it specifically states:

“Current and future land [use] such as…sport hunting and fishing should not be restricted due to the designation of the nonessential experimental population of California Condors […] the [US Fish and Wildlife] Service does not intend to request modifications or restrictions to the current hunting regulations anywhere […] in the experimental population area” [46].

This final ruling may make hunters more supportive of California Condor conservation by assuring hunters the federal government has no intention to regulate hunting or shooting on account of the experimental, non-essential population of California Condors. Further, this final ruling may help hunters cognitively and affectively dissociate the issue of conservation from perceived attempts as limiting rights of gun ownership. Additionally, agencies may underscore their support of shooting sports and promulgation of shooting ranges to reaffirm their commitment to recreational shooting, and, by extension, the hunting heritage.

The intent of this study was to comprehend and anticipate the behaviors of hunters regarding nonlead ammunition in the American Southwest. Plainly, nonlead ammunition voucher programs induce a higher rate of voluntary compliance. This study also offers insight into which factors play a role in the ammunition use decision, which is necessary to encourage future voluntary compliance. Barriers to using nonlead ammunition primarily were obtaining it (particularly in the desired caliber) and its higher cost. Conversely, those who did use nonlead ammunition did so because of the AGFD request and a personal desire to help California Condor (and scavengers more generally) conservation. Conservation of California Condors [13,14], and other wildlife more generally [2, 11, 12], will be improved if state wildlife agencies continue to collaborate with ammunition manufacturers to improve market availability of nonlead ammunition, partner with sporting goods retailers to produce a universal label to facilitate the decision to use nonlead ammunition should hunters elect to do so, and encourage hunters to continue the hunting tradition through wildlife conservation of all species.

## Supporting Information

S1 File Questionnaire used to measure hunters’ attitudes toward use of nonlead ammunition. A copy of the questionnaire instrument is provided for meta-analysis purposes. Page one consists of the cover letter that explains the study intent, participants’ rights and expectations, and the method to gain further information regarding the study. Page two contains survey items regarding general hunting and lead-specific behaviors. Respondents are split according to their response to their use of lead or nonlead ammunition and asked topic specific questions.
(PDF)

## References

[1] Diters RW, Nielsen SW (1978) Lead poisoning of raccoons in Connecticut. Journal of Wildlife Diseases 14(2):187–192. 650783 10.7589/0090-3558-14.2.187

[2] Haig SM, D'Elia J, Eagles-Smith CA, Fair JM, Gervais J, Herring G, et al (2014) The persistent problem of lead poisoning in birds from ammunition and fishing tackle. Condor 116(3):408–428.

[3] Blus LJ, Henny CJ, Hoffman DJ, Sileo L, Audet DJ (1999) Persistence of high lead concentrations and associated effects in Tundra Swans captured near a mining and smelting complex in northern Idaho. Ecotoxicology 8:125–132.

[4] Samuel MD, Bowers EF (2000) Lead exposure in American Black Ducks after implementation of non-toxic shot. Journal of Wildlife Management 64:947–953.

[5] Fair JM, Myers OB (2002) The ecological and physiological costs of lead shot and immunological challenge to developing Western Bluebirds. Ecotoxicology 11:199–208. 12092753 10.1023/a:1015474832239

[6] Brennan LA (1991) How can we reverse the Northern Bobwhite population decline? Wildlife Society Bulletin 19: 544–555.

[7] Larsen RT, Flinders JT, Mitchell DL, Perkins ER (2007) Grit size preferences and confirmation of ingested lead pellets in Chukars (Alectoris chukar). Western North American Naturalist 67:152–155.

[8] Ferrandis P, Mateo R, López-Serrano FR, Martínez-Haro M, Martínez-Duro E (2008) Lead shot exposure in Red-legged Partridge (Alectoris rufa) on a driven shooting estate. Environmental Science and Technology 42:6271–6277. 18767698 10.1021/es800215y

[9] Craighead D, Bedrosian B (2008) Blood lead levels of Common Ravens with access to big-game offal. Journal of Wildlife Management 72:240–245.

[10] Kelly TR, Bloom PH, Torres SG, Hernandez YZ, Poppenga RH, Boyce WM, et al (2011) Impact of the California lead ammunition ban on reducing lead exposure in Golden Eagles and Turkey Vultures. PLoS ONE 6(4):e17656 10.1371/journal.pone.0017656 21494329 PMC3071804

[11] Bedrosian B, Craighead D, Crandall R (2012) Lead exposure in Bald Eagles from big game hunting, the continental implications and successful mitigation efforts. PLoS ONE 7(12):e51978 10.1371/journal.pone.0051978 23284837 PMC3526477

[12] Harmata AR, Restani M (2013) Lead, mercury, selenium, and other trace elements in tissues of Golden Eagles from southwestern Montana, USA. Journal of Wildlife Diseases 49: 114–124. 10.7589/2012-01-004 23307377

[13] Finkelstein ME, Doak DF, George D, Burnett J, Brandt J, Church M, et al (2012) Lead poisoning and the deceptive recovery of the critically endangered California condor. Proc Natl Acad of Sci 109: 11449–11454. 22733770 10.1073/pnas.1203141109 PMC3396531

[14] Walters JR, Derrickson SR, Fry MD, Haig SM, Marzluff JM, Wunderle JM (2010) Status of the California condor (Gymnogyps Californianus) and efforts to achieve its recovery. Auk 127:969–1001.

[15] Snyder N, Snyder H (2000) The California Condor: A saga of natural history and conservation. San Diego, California, Academic Press, 410 pp.

[16] Rideout BA, Stalis I, Papendick R, Pessier A, Puschner B, Finkelstein ME, et al (2012) Patterns of mortality in free-ranging California Condors (Gymnogyps californianus). J Wildl Dis 48: 95–112. 22247378 10.7589/0090-3558-48.1.95

[17] Sullivan KA, Sieg R, Parish C (2009) Arizona’s efforts to reduce lead exposure in California Condors In: Mee A Hall LS. California Condors in the 21st century. Lancaster, Pennsylvania: Cadmus Communications pp. 109–121.

[18] Sieg R, Sullivan KA, Parish CN (2009) Voluntary lead reduction efforts within the northern Arizona range of the California Condor In: Watson RT Fuller M Pokras M, Hunt WG. Ingestion of lead from spent ammunition: Implications for wildlife and humans. Boise Idaho: The Peregrine Fund pp. 341–349.

[19] Wright TD, Peddicord RK (11 6 2007). Summary of science for ammunition as the source of lead in California Condors. The National Rifle Association Available: http://www.cspinet.org/new/pdf/nra_-_lead_ammo_-_s._recce.pdf. Accessed 8 August 2013.

[20] Hunt WG, Burnham W, Parish CN, Burnham KK, Mutch B (2006) Bullet fragments in deer remains: Implications for lead exposure in avian scavengers. Wildl Soc Bull 34:167–170.

[21] Hunt WG, Parish CN, Farry SC, Lord TG, Sieg R (2007) Movements of introduced California Condors in Arizona in relation to lead exposure In: Mee A Hall LS. California Condors in the 21st century. Lancaster, Pennsylvania: Cadmus Communications pp. 109–121.

[22] Church ME, Gwiazda R, Risebrough RW, Sorenson K, Chamberlain CP, Farry S, et al (2006) Ammunition is the principal source of lead accumulated by California Condors re-introduced to the wild. Environ Sci Technol 40:6143–6150 17051813 10.1021/es060765s

[23] Avery D, Watson RT (2009) Regulation of lead-based ammunition around the world In: Watson RT Fuller M Pokras M, Hunt WG. Ingestion of lead from spent ammunition: Implications for wildlife and humans. Boise Idaho: The Peregrine Fund pp.161–168.

[24] USFWS (2013) US Fish and Wildlife Services California Condor Recovery Program Monthly status report. Available: http://wwwfwsgov/hoppermountain/CACORecoveryProgram/CurrentStatus.html. Accessed 14 Jan 2014.

[25] Grund MD, Cornicelli L, Carlson LT, Butler EA (2010) Bullet fragmentation and lead deposition in white-tailed deer and domestic sheep. Human-Wildlife Interactions 4:257–265.

[26] Responsive Management (2003) Hunters’ knowledge of and attitudes towards threats to California Condors. Report to the US Fish and Wildlife Service, California Condor Recovery Team, and California Condor Lead Exposure Reduction Committee. Available: http://wwwresponsivemanagement.com/download/reports/AZ_CaliforniaCondor_Report.pdf. Accessed 5 April 2010.

[27] Seng PT (2006) Non-lead ammunition program hunter survey. Final report to the Arizona Game and Fish Department. Available: http://www.azgfd.gov/w_c/documents/AmmoSurveyFINALReport2-23-06_000.pdf. Accessed 5 April 2010

[28] Friend M, Franson JC, Anderson WL (2009) Biological and societal dimensions of lead poisoning in birds in the USA In: Watson RT Fuller M Pokras M, Hunt WG. Ingestion of lead from spent ammunition: Implications for wildlife and humans. Boise Idaho: The Peregrine Fund pp.34–60.

[29] Hunt WG, Parish CN, Orr K, Aguilar RF (2009) Lead poisoning and the reintroduction of the California Condor in Northern Arizona. J Avian Med Surg 23: 145–150. 19673462 10.1647/2007-035.1

[30] Green RE, Hunt WG, Parish CN, Newton I (2008) Effectiveness of action to reduce exposure of free-ranging California Condors in Arizona and Utah to lead from spent ammunition. PLoS ONE 3: 240–253. 10.1371/journal.pone.0004022 PMC2603582 19107211

[31] Green RE, Hunt WG, Parish CN, Newton I (2009) Effectiveness of action to reduce exposure of free-ranging California Condors in Arizona and Utah to lead from spent ammunition In: Watson RT Fuller M Pokras M, Hunt WG. Ingestion of lead from spent ammunition: Implications for wildlife and humans. Boise Idaho: The Peregrine Fund pp.240–253.

[32] Putnam RD (2000) Bowling alone: The collapse and revival of American Community. New York: Simon & Schuster 544 p.

[33] Prukop J, Regan RJ (2005) The value of the North American model of wildlife conservation: An International Association of Fish and Wildlife Agencies position. Wildl Soc Bull 33: 374–377

[34] Dillman D, Smyth J, Christian L (2009) Internet, Mail, and Mixed-Mode Surveys: The Tailored Design Method. New York: Wiley 528 p.

[35] Vaske JJ (2008) Survey research and analysis: Applications in parks, recreation, and human dimensions. Pennsylvania: Venture Publishing Inc 635 p.

[36] Chase, L (2012) Reducing Lead on the Landscape. Report to Arizona Game and Fish Department. Available: www.azgfd.gov/California Condors. Accessed 31 Jul 2013.

[37] Trinogga A, Fritsch G, Hofer H, Krone O (2012). Are leadfree hunting rifle bullets as effective at killing wildlife as conventional lead bullets? A comparison based on wound size and morphology. Science of the Total Environment 443: 226–232. 10.1016/j.scitotenv.2012.10.084 23186634

[38] Thomas VG (2013) Lead-free hunting rifle ammunition: Product availability, price, effectiveness, and role in global wildlife conservation. AMBIO 42:737–745. 10.1007/s13280-012-0361-7 23288616 PMC3758820

[39] Petty RE, Cacioppo JT (1986) Communication and persuasion: Central and peripheral routes to attitude change. New York: Springer-Verlag 264p.

[40] Epps CW (2014) Considering the switch: Challenges of transitioning to non-lead hunting ammunition. The Condor 116(3):429–434.

[41] Schroeder SA, Fulton DC, Penning W, Doncarlos K (2012). Using persuasive messages to encourage hunters to support regulation of lead shot. Journal of Wildlife Management 76:1528–1539.

[42] Ajzen I (1991) The Theory of Planned Behavior. Organizational Behavior and Human Decision Processes 50:179–211.

[43] Ajzen I, Fishbein M (1980) Understanding attitudes and predicting social behavior. New Jersey: Prentice Hall 278p.

[44] Vaske JJ, Donnelly MP (2007) Public knowledge and perception of the desert tortoise. Report for the National Park Service. Available: http://www.dmg.gov/documents/STDY_Pblc_Knwldge_Prcptns_DT_VaskeJ_092007.pdf. Accessed 16 Dec 2013.

[45] Pokras MA, Kneeland MR (2008) Lead poisoning: Using transdisciplinary approaches to solve an ancient problem. EcoHealth 5:379–385. 10.1007/s10393-008-0177-x 19165554

[46] USFWS (1996) Final rule- Endangered and threatened wildlife and plants: Establishment of a nonessential experimental population of California Condors in Northern Arizona. Federal Register 67: 54044–54060.

