# Supplementary material for: Reducing Lead on the Landscape: Anticipating Hunter Behavior in Absence of a Free Nonlead Ammunition Program
Source: PLoS One. 2015 Jun 26;10(6):e0128355. doi: 10.1371/journal.pone.0128355 (PMC4482685; doi:10.1371/journal.pone.0128355)
Supplement: S1 File — A copy of the questionnaire instrument is provided for meta-analysis purposes. Page one consists of the cover letter that explains the study intent, participants’ rights and expectations, and the method to gain further information regarding the study. Page two contains survey items regarding general hunting and lead-specific behaviors. Respondents are split according to their response to their use of lead or nonlead ammunition and asked topic specific questions. (PDF) [file pone.0128355.s001.pdf]

THE STATE OF ARIZONA
GAME AND FISH DEPARTMENT
5000 W. CAREFREE HIGHWAY
PHOENIX, AZ 85086-5000
(602) 942-3000 • WWW.AZGFD.GOV

GOVERNOR
JANICE K. BREWER
COMMISSIONERS
CHAIRMAN, NORMAN W. FREEMAN, CHINO VALLEY
JACK F. HUSTED, SPRINGERVILLE
J.W. HARRIS, TUCSON
ROBERT E. MANSELL, WINSLOW
KURT R. DAVIS, PHOENIX
DIRECTOR
LARRY D. VOYLES
DEPUTY DIRECTORS
GARY R. HOVATTER
BOB BROSCHEID

<Address block>

Dear <First>

A short time ago, we sent you a notification postcard regarding a study that we were doing to measure the satisfaction of your 2011 hunt in unit 9 or 10. The goal of this study is to measure the following:

1. the outcome of your hunt
2. the steps you may have taken to reduce the amount of lead available to wildlife

Information collected in this survey will be used to inform future management decisions in the area as well as demonstrate the success of Arizona's hunters as the original conservationists.

Participation in this survey is voluntary and we ask that you take a few minutes of your time to answer the questions. Game management units 9 and 10 are the only areas we are studying this closely and you are one of a select few that has been randomly selected to participate, so your response in crucial.

Each survey has a random identification number, yours is <UID>. We use this number for the following reasons:

1. **To save hunting and fishing dollars**. So we do not send you multiple surveys, we erase this number from our list as soon as you return this survey. We save substantial hunting and angling dollars in postage/processing costs by using this number.
2. **To maintain confidentiality.** This number is destroyed once the study is complete. Your contact, hunting, or your draw information will never be associated with your responses, nor will it be sold.
3. **To ensure quality**. Data entry errors or data irregularities can be quickly identified by using this number, again saving costs.

The Department would be happy to answer any questions you might have regarding this survey. Please feel free to contact me by phone or email (details provided below). Thank you very much for your assistance.

Sincerely,

Kathy Sullivan
Region 2 Wildlife Program
Arizona Game and Fish Department
928-214-1249
KSullivan@AZGFD.GOV

# 2011 Unit 9 and 10 Hunter Survey

| | | | |
|---|---|---|---|
| 1. Did you go hunting in either unit 9 or 10 during your 2011 hunt? | Y | N | |
| *If yes*, How many days were you in the field? | | | _________days hunting<br>_________days scouting |
| 2. Did you harvest an animal on your hunt? | Y | N | |
| 3. Had you hunted in this hunting unit before your 2011 hunt? | Y | N | What year?______ |
| 4. Before your hunt, did you receive a letter from AZ Game and Fish about voluntary lead reduction efforts? | Y | N | |
| 5. Did you use non-lead ammunition on your hunt? | Y | N | |

*Fill out this section if you answered "**YES**" to question 5*

| | Strongly Disagree | Disagree | Neither | Agree | Strongly Agree |
|---|---|---|---|---|---|
| Based on my experience, I would use it again in the future. | 1 | 2 | 3 | 4 | 5 |
| Would you recommend it to fellow hunters | 1 | 2 | 3 | 4 | 5 |

| What factors were important to you while making your decision to use non-lead ammunition? | Not important at all | | | | Extremely Important |
|---|---|---|---|---|---|
| I already used it | 1 | 2 | 3 | 4 | 5 |
| I read/heard about its performance | 1 | 2 | 3 | 4 | 5 |
| Game & Fish asked me to | 1 | 2 | 3 | 4 | 5 |
| To help condor recovery efforts | 1 | 2 | 3 | 4 | 5 |
| I like the performance of nonlead ammunition | 1 | 2 | 3 | 4 | 5 |
| Other__________________ | 1 | 2 | 3 | 4 | 5 |

Which brand of non-lead ammunition did you use?

- ◻ Barnes TSX
- ◻ Nosler E-tip
- ◻ Hornady BMX
- ◻ Other ________________________________

*Fill out this section if you answered "**NO**" to question 5*

| | Strongly Disagree | Disagree | Neither | Agree | Strongly Agree |
|---|---|---|---|---|---|
| It costs too much for me to use | 1 | 2 | 3 | 4 | 5 |
| It wasn't available in my caliber | 1 | 2 | 3 | 4 | 5 |
| It is too difficult to find in stores | 1 | 2 | 3 | 4 | 5 |
| It doesn't perform as well as lead | 1 | 2 | 3 | 4 | 5 |
| I forgot to use it | 1 | 2 | 3 | 4 | 5 |
| I'm not convinced lead poisoning is a problem for condors | 1 | 2 | 3 | 4 | 5 |
| Other_________________ | 1 | 2 | 3 | 4 | 5 |

What would have convinced you to use non-lead ammunition on your hunt?

- ◻ If it cost the same as lead
- ◻ If it were provided for free
- ◻ If it performed better
- ◻ If it were available in my caliber
- ◻ If there were more information available
- ◻ Nothing
- ◻ Other__________________________

If you harvested an animal with lead ammunition, did you remove the gut pile from the field? ◻ Yes ◻ No

*Now, would like to finish by asking you just a few questions about yourself to help us analyze the data*

What is your age? _______

What is your Zip code? _________________

*Feel free to provide additional comments on the reverse side of this survey.* **<UID>**
